# Duplication of chromosome 1q32.1q44: presented with ventriculomegaly and transient myeloproliferative disorder of the newborn

**DOI:** 10.1515/crpm-2024-0031

**Published:** 2025-05-28

**Authors:** Medha Goyal, Malgorzata Joanna Nowaczyk, Vicky Breakey, Elizabeth McCready, Ipsita Goswami

**Affiliations:** Division of Neonatology, Department of Pediatrics, McMaster Children’s Hospital, McMaster University, Hamilton, Canada; Division of Clinical Pathology and Molecular Medicine, McMaster University, Hamilton, Canada; Department of Pediatrics, McMaster Children’s Hospital, McMaster University, Hamilton, Canada

**Keywords:** duplication 1q, translocation, prenatal ventriculomegaly, encephalomalacia, lymphoproliferative disorder

## Abstract

**Objectives:**

Partial trisomy of chromosome 1 has been reported following unbalanced translocations with partial monosomies of other chromosomes and rarely as a pure partial duplication. We aim to discuss partial trisomy 1q with cytogenetics and describe our findings of this uncommon chromosomal aneuploidy.

**Case presentation:**

A male term neonate presented with antenatal ventriculomegaly and early fetal growth restriction. He was dysmorphic at birth and his postnatal course was complicated by transient myeloproliferative disorder, neonatal seizures, skin rash, conjugated hyperbilirubinemia, and milk protein allergy. Etiological work-ups including congenital infections, immunological disorders, and inborn error of metabolisms were negative. The findings of transient myeloproliferative disorder in association with partial 1q trisomy which have not been previously described in the literature, raise the possibility of abnormal vasculature of generalized nature, resulting in cutis marmorata, signs of intestinal inflammation, and abnormal cerebral vascular supply.

**Conclusions:**

This case study highlights the importance of pooling cases with similar locations of duplication, segment size, and related chromosomal deficiency together to understand distinct clinical phenotypes.

## Introduction

Partial trisomy of chromosome 1 has been reported in literature following unbalanced translocations with partial monosomies of other chromosomes and rarely as a pure partial duplication [1]. In this report, a case of duplication of 1q32 → qter due to an unbalanced translocation between chromosomes 1 and 10 is presented. The translocation breakpoints (1q32 and 10q26.3) result in partial trisomy of 1q, but no detectable loss of genes from 10q26.3, suggesting that the observed phenotype is likely associated with the extra 1q material rather than loss of chromosome 10 material.

Pure 1q partial trisomies are rare in the literature. A literature search found one single case reported in 1978 of a term female infant with a similar translocation [1]. By comparing the clinical findings in the current case to those reported previously, we present further evidence of the clinical course of this rare chromosome abnormality.

## Case presentation

This male infant was born to a 31-year-old G3SA1P1L1 mother from a planned spontaneous pregnancy. There was no history of hypertension or diabetes nor maternal exposures to medications, smoking, alcohol, or recreational drugs. The mother had COVID-19 and several upper respiratory tract infections during early pregnancy. She had a normal, non-invasive prenatal screen. The parents were non-consanguineous, of Polish-German and Guyanese-French-Canadian ancestry.

At 20 weeks of gestation, a sonographic evaluation showed the proximal aorta (AO) larger than the main pulmonary artery (PA) and expected fetal weight (EFW) at the 3rd centile, resulting in a referral to our center. Sonographic evaluation at 23 + 4 showed EFW at the 18th percentile, an asymmetry between the PA and AO, with the PA being smaller than the AO and measuring smaller than expected, and post-valvular dilation of the left ventricular outflow tract. Fetal echocardiogram at 26 + 4 weeks gestation showed a dilated ascending aorta, abnormal systolic flow seen in the ascending and transverse arch, and main PA at the bifurcation of the branch PAs, with good biventricular function. The sonogram at 28 + 2 weeks showed bilateral cerebral ventriculomegaly (left side measuring 16 mm, right side measuring 14.5 mm) with bilateral dangling choroid plexus; a Blake pouch cyst was also noted. A possible sandal gap appearance on the left foot was reported. The parents declined genetic testing throughout the pregnancy.

The infant was born at 37 + 1 weeks via induced vaginal delivery. Birth weight was 2.26 kg (5 %), length was 43.5 cm (2 %), head circumference was 31.5 cm (10 %). Apgar scores were 8 and 8 at 1 and 5 min. He was initially breathing well on room air, but at around 9 min of life started to have tachypnea and respiratory distress, necessitating continuous positive airway pressure therapy.

**Figure 1:**
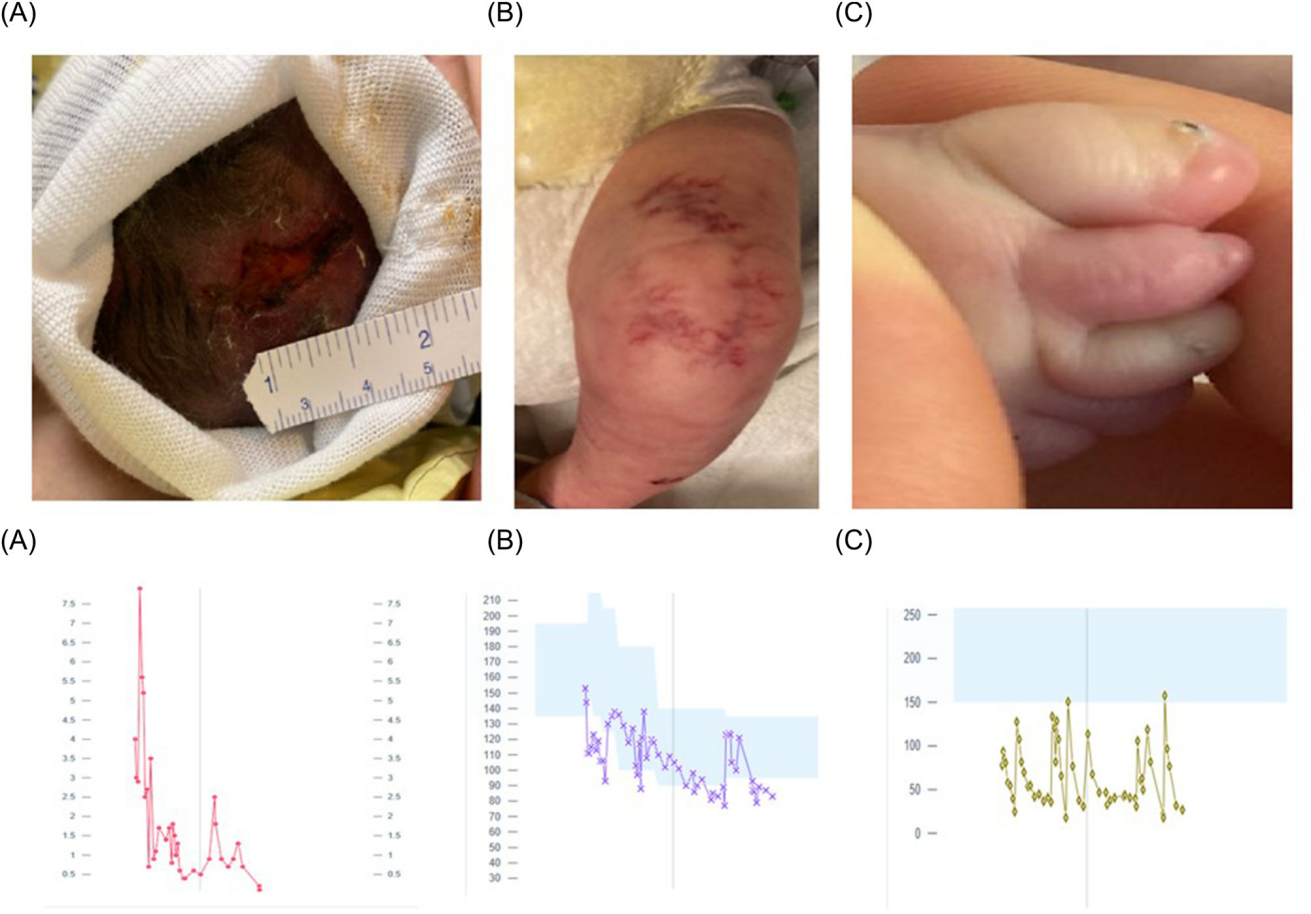
*Top panel:* clinical images demonstrating (A) cutis aplasia, (B) cutis marmorata telangiectasia, and (C) foot with 2nd and 3rd toe syndactyly and rudimentary fifth toe with dysplastic toenails in all toes. *Bottom panel:* Trend of hematological parameters throughout hospitalization: (A) blast cells; (B) hemoglobin; and (C) platelets.

On physical examination on day one of life, he had low-set ears and a flat nasal bridge. He had a high-arched palate. There was cutis aplasia of the scalp at the vertex (Figure 1A) and cutis marmorata of the extremities (both knees and over the left arm) (Figure 1B). External genitalia was a normal male with bilaterally descended testes. He had small, dysplastic toes (5 digits on both feet, but 5th digits very small) and dysplastic toenails (Figure 1C). His hands were held in a clenched-fisted position with his thumb tucked in and crossed over his fingers.

On day 1 of life, he was found to have leukocytosis with high peripheral blast count and thrombocytopenia (Figure 1 *(bottom panel)*)*.* On flow cytometry, blast cells were Cluster of differentiation [CD] 45+, CD 34−, CD117+, CD19−, heterogeneous CD 33+, CD 13−, dim CD11b+, CD 15−, CD64−, CD14−, CD7+ indicative of helper or suppressor T-cells immunophenotype and polyclonal B cells. A fluctuating pattern of peripheral monoblasts and thrombocytopenia continued for the initial 2 months of life. Differential included acute myeloid leukemia (CD 117+, CD 33+, CD 61+, CD71+ but MPO−) and acute promyelocytic leukemia (CD 34−, HLA-DR−, CD117+, CD33+) were considered. Testing for t(15; 17) ruled out acute promyelocytic leukemia and common fusion translocations (PML::RARA, RUNX1::RUNX1T1, CBFB::MHY11, and BCR::ABL1). Next-generation sequencing of DNA extracted from peripheral blood was negative for genetic variants in 25 myeloid malignancy-associated genes including NPM1 and FLT3). The hematologic abnormalities were concluded to be a transient myeloproliferative process associated with the underlying chromosomal anomaly. He required multiple packed cells and platelet transfusions in the first three months of life. The peripheral blasts resolved at approximately 3 months of age.

**Figure 2:**
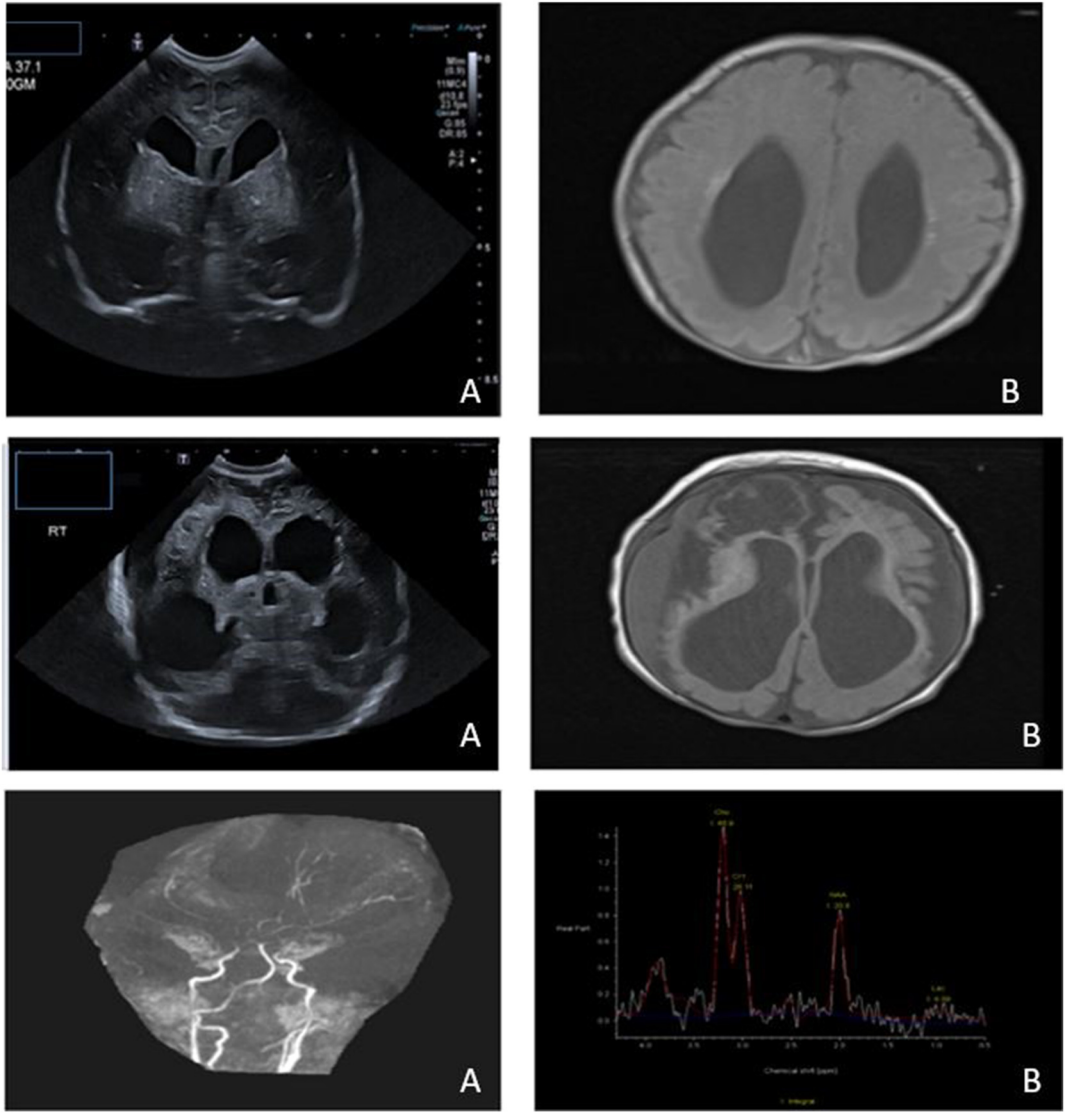
Serial neuroimaging. *Top panel:* (A) cranial ultrasound image on day 1 of life showing ventriculomegaly with colpocephaly (B) MRI brain on day 4 of life: coronal T1 images with dilatation of lateral ventricles with colpocephalic configuration (right > left) with multiple hyperintense foci in periventricular white matter *middle panel:* (A) cranial ultrasound at 3 months showing progressive ex-vacuo dilatation of ventricles and evolving cystic encephalomalacia (B) follow-up MRI at 3 months of life shows significant diffuse volume loss of cerebral white matter with intraparenchymal multicystic changes marked in the right frontal lobe. *Bottom panel:* (A) time of flight MRA study showed severe attenuation of the supra clinoid segments of internal carotid arteries and bilateral M1 and P1 segments, absent flow in the right anterior cerebral artery and small left anterior cerebral artery. (B) MR spectroscopy over the left basal ganglia with low NAA and small lactate peak.

Cranial sonography [US] on day of life (DOL) 1 confirmed supratentorial ventriculomegaly with colpocephaly, periventricular, and basal ganglia calcification bilaterally (Figure 2A). Magnetic resonance imaging (MRI) brain on DOL4 revealed dilated lateral ventricles (posterior > anterior), calcification of thalami, basal ganglia, and left periventricular white matter (Figure 2B).

Postnatal echocardiography on DOL1 showed normal biventricular function and a moderate patent ductus arteriosus, which was obliterated on follow-up echocardiography at 24 h.

Evaluation for TORCH infections, including toxoplasmosis, rubella, syphilis, herpes, and cytomegalovirus was negative. Ophthalmological and hearing assessments were normal. Newborn screening, evaluation for various inborn errors of metabolism, and skeletal survey were also normal.

He developed seizures at DOL 24, with bilateral eye twitching and concurrent focal onset seizures on electroencephalogram (EEG). Seizures were controlled with phenobarbitone and levitiracetam. EEG also revealed a very dysmature background with a potential for evolution into an epileptiform pattern. These seizures aligned with evolving cystic encephalomalacia changes in serial cranial ultrasound (Table 1). Subsequent cranial ultrasounds demonstrated an increase in ventricular dimensions with cortical mantle thinning, cystic encephalomalacia changes, and an increase in extra-axial cerebrospinal fluid space by the end of the second month of life (Figure 2 (*middle panel*)). The change in ventricular dimension was not associated with signs of raised intracranial tension such as sutural separation, agitation, or bulged fontanelle hence, was in keeping with ex-vacuo dilatation secondary to white matter loss. An MRI at three months found significant cerebral white matter loss with evolving cystic encephalomalacia and increased ventricular dimensions (Figure 2 (*middle panel*)). On MR angiography severe attenuation of the supraclinoid segments of internal carotid arteries with absent flow in the right anterior cerebral artery and small left anterior cerebral artery was seen (Figure 2 (*bottom panel*)).

**Table 1:** Trajectory of changes in ventricular dimension on serial cranial ultrasound.

	Right anterior horn width, in mm	Left anterior horn width, in mm	Third ventricle, in mm	Right ventricular index, in mm	Left ventricular index, in mm
Day 1	9.7	10.2	2.5	19	17
Day 3	8.6	8.3	2.5	20	17
Day 14	6.4	7.5	2.5	20	17
Day 21	9.5	10.3	3.0	21	17
Day 25	16.8	14.9	3.0	21	18
Day 35	10.3	12.4	2.5	22	18
Day 50	9.9	16.0	3.1	22	19
Day 56	14.0	17.9	3.0	22	23
Day 72	19.9	23.3	3.0	22	23.5
Day 78	18.1	19.0	3.0	21.7	25.8

At the age of three weeks, he developed bloody stools which were in keeping with cow’s milk protein allergy and responded well to switching from breast milk to elemental formula. He also developed transient conjugated hyperbilirubinemia in the fourth week of life. Tests showed normal liver enzymes and abdominal ultrasound; a hepatobiliary iminodiacetic acid scan ruled out biliary atresia.

He remained stable without respiratory support, able to do non-nutritive sucking at the breast. Despite a normal neurological status at birth, including appropriate alertness, muscle tone, and active and symmetrical movements in all limbs, he showed signs of decreased alertness, increased appendicular tone, jerky movements upon awakening, and difficulty swallowing and handling oral feeds by 3 months of age. By the time of his discharge home at 3.5 months of life, he was feeding with a nasogastric tube for feeding. He weighed 5,260 g (23rd percentile) and had a head circumference of 35.8 cm (<1st percentile).

In the months that followed, the baby’s care has been coordinated by the palliative care team. He remains alive at nine months of age. He recently had a G tube placed to support his nutrition. From a hematology perspective, his transfusion dependency settled, but he continues to have thrombocytopenia (platelets 30–70 × 10^–9^/L). His anemia improved but recurred with intercurrent illness at 8 months of age. No bone marrow testing has been done to focus on supportive care.

## Results

Chromosomal microarray testing was performed using the Affymetrix Cytoscan HD Microarray assay (ThermoFisher Scientific). It showed a large copy number gain resulting in an extra copy of material from the terminus of chromosome one distal to a breakpoint at 1q32.1; arr[GRCh37] Xq28(148,833,016_149,105,766)x0, 1q32.1q44(204,011,992_248,753,203)x3. This pathogenic copy number gain detected was of size 44,741 kb resulting in duplication of over 300 protein-coding genes. An Xq28 copy number loss was also detected (approximately 278 kb in size), but none of the genes from this region (HSFX1, MAGEA9, MAGEA8, HSFX4 and EOLA2) are known to be dosage-sensitive, and the deletion was interpreted as a variant of uncertain significance. G-banded metaphase chromosomes (at a resolution of 450) showed that the extra 1q material is located on the long arm of one chromosome 10. The material from chromosome 1 (region 1q32.1-1qter) had replaced the telomeric end of chromosome 10 (region 10q26.3). Karyotype was 46, XY, der(10)t(1;10)(q32.1;q26.3). Despite the detection of a derivative chromosome 10 in metaphase cells, the absence of chromosome 10 copy number losses, as assessed by chromosome microarray, suggests intact protein-coding sequences. Parents did not opt for any genetic testing and it was therefore not possible to determine whether the observed genetic variants are inherited from the parents or occur *de novo*.

## Discussion

We describe the clinical presentation of a case of partial trisomy of chromosome 1q who presented with fetal ventriculomegaly, growth restriction, transient myeloproliferative disorder, neonatal seizures with neurodevelopmental regression, and cystic encephalomalacia. Based on our literature review, this is likely the second reported case of partial trisomy 1q resulting from an unbalanced translocation between chromosomes 1 and 10 with breakpoints at 1q32.1 and 10q26.3 without any loss of protein-coding sequences from chromosome 10. As chromosome one is the largest of the human chromosomes, comprising 8 % of the whole genetic material and nearly 3,000 genes, a complete trisomy remains incompatible and leads to spontaneous abortions [2]. In the literature, there are few reports of trisomy of segments of the long arm of chromosome 1, which often occurs due to unbalanced translocations with partial monosomies of other chromosomes and rarely as a pure partial duplication [1], [3], [4], [5], [6].

The hot spots of the distal third of chromosome one involved in long-arm trisomy are q23, q24, q25, q32, and q42 [5]. In cases of unbalanced translocation, associated deficiencies in the reciprocal chromosome are often confined to the telomeric part of the p or q arm. However, an unbalanced translocation in chromosome 10 (region 10q26.3) is extremely rare, and in our case, no genes from chromosome 10 were deleted by this translocation. In a report by Bonfante et al. [1] of a term female infant with partial trisomy of the long chromosome of chromosome 1 with an elongated chromosome 10 on karyotyping. The breakpoints were found in the proximal part of band 1q32 and on chromosome 10 at the distal part of band 10q26. As no chromosomal microarray was available, it is not possible to comment on the impact of this translocation on protein-coding sequences. The father was found to carry the unbalanced translocation; however, in our case, the parental carrier state remained unestablished as the parents opted out of the genetic testing, which is an important limitation as it limits our understanding of whether the chromosomal abnormality was inherited or *de novo* [1].

Clinical suspicion of distal partial trisomy 1q at birth may stem from overlapping symptoms such as mild facial dysmorphism, limb defects, genitourinary, cardiac, cranial anomalies, and growth retardation. Distinctive phenotype can result from various facial and limb abnormalities, such as macrocephaly with sutural splaying, microcephaly, trigonocephaly, mid-face hypoplasia, microphthalmia, hypertelorism, deep-set eyes, down-slanting palpebral fissures, short nasal bridge, microstomia, long philtrum, high arched palate, cleft palate/cleft lip, and low set ears. Limb defects may include syndactyly, clinodactyly, nail dysplasia, long tapering fingers, simian crease, proximally impacted thumbs, and sandal gaps [2], [3], [4], [5]. Our index case was typical for craniofacial and limb features within this phenotype.

Cardiac malformations in partial 1q trisomy are linked to duplication sites 1q23 and 1q25, crucial for aortopulmonary septum development. Duplications in this segment often cause aortic or pulmonary hypoplasia and arch abnormalities [1], 5]. Although we were concerned about potential heart defects such as aortopulmonary window defects or bicuspid aortic valve, postnatal echocardiography showed that the infant did not have any congenital cardiac anomalies. Gastrointestinal anomalies associated with absent gallbladder and common bile duct stenosis have been described. However, we found evidence of transient cholestasis without any structural gastrointestinal lesions. Other gastrointestinal anomalies reported are esophageal atresia, pyloric, and duodenal stenosis [1], [3], [4], [5], [6]. In contrast to other reports, we did not discover any abnormalities in the genital or urinary systems [1], 5], 6].

A striking systemic involvement not reported previously was the occurrence of transient myeloproliferative response seen in our case, though the association of partial trisomy 1q with acute myeloid leukemia in two adult patients has been reported [7]. However, in neonates, transient myeloproliferative disorder has been classically reported with Trisomy 21 with GATA-1 mutation [8]. Furthermore, although the genetic abnormality was similar, the case described by Bonfante et al. [1] had a distinct set of systemic issues, including defects in the atrial and ventricular septa, hypoplastic mitral and tricuspid valves, and stenosis of the ileum and colon [1]. These phenotypic differences may be partially related to differences in the translocated segments at the genomic level that might not have been discerned with karyotyping alone. Moreover, the possibility of Xq28 contributing to the phenotype in our case can not be ruled out based on the available evidence. This emphasizes the potential significance of assigning cases with similar locations of duplication, segment size, and related chromosomal deficiency in establishing these distinct clinical phenotypes.

In our patient, by 2 months of age, the progressive nature of encephalopathy was clinically obvious, and unfortunately, the trajectory aligned with previous reports describing severe neurological impairment [6], 9]. The progressive trajectory of white matter loss was unexplained. The potential etiologies of the progressive white matter loss may include intrinsic systemic vasculopathy or vascular narrowing leading to chronic hypoxia and white matter loss of the brain. In our case, we speculate the abnormal vasculature was more generalized, resulting in cutis marmorata, signs of intestinal inflammation, and abnormal cerebral vascular supply. Vascular injuries with thrombotic anomaly and vasculopathy have pathophysiology of these vasculopathies currently remains unknown.

Life expectancy and quality are variable and often depend upon systemic abnormalities. In a previous similar report, the neonate passed away at 26 days [1]. Our infant was discharged at 3.5 months and is alive at nine months at the time of reporting, albeit severely neurologically impaired. The goals of care at discharge focussed on good quality of life with minimal pain, with preventive strategies to maintain health while ensuring his comfort as a primary focus.

## Conclusions

This case adds to the existing knowledge of this chromosomal duplication and highlights transient myeloproliferative disorder as previously unrecognized phenotypes that can occur in association with partial 1q trisomy. Additional case reports of patients with similar 1q duplications are important to further elucidate the phenotypic spectrum associated with imbalances of this chromosome region.

## Learning points

1. Partial trisomy of chromosome one often follows unbalanced translocations with partial monosomies of other chromosomes and rarely as a pure partial duplication.
2. Pure 1q partial trisomies are an extremely rare cause of antenatal ventriculomegaly.
3. The constellation of findings of mild facial dysmorphism, limb defects, genitourinary, cardiac, cranial anomalies, and growth retardation should raise clinical suspicion of distal partial trisomy 1q.

## What is new?

1. We describe findings of transient myeloproliferative disorder as a previously unrecognized phenotype associated with partial 1q trisomy in neonates.
2. We highlight the possibility of generalized abnormal vasculature being a pathogenic process in partial trisomy 1q, resulting in cutis marmorata, signs of intestinal inflammation, and abnormal cerebral vascular supply.

## References

[1] Bonfante A, Stella M, Rossi G (1978). Partial trisomy of the long arm of chromosome 1 due to a familial translocation t (1; 10)(q32; q26). Hum Genet.

[2] Sayers EW, Beck J, Bolton EE, Bourexis D, Brister JR, Canese K (2021). Database resources of the National Center for biotechnology information. Nucl Acids Res.

[3] Nowaczyk MJ, Bayani J, Freeman V, Watts J, Squire J, Xu J (2003). De novo 1q32q44 duplication and distal 1q trisomy syndrome. Am J Med Genet.

[4] Balasubramanian M, Barber JC, Collinson MN, Huang S, Maloney VK, Bunyan D (2009). Inverted duplication of 1q32. 1 to 1q44 characterized by array CGH and review of distal 1q partial trisomy. Am J Med Genet.

[5] Johnson VP (1991). Duplication of the distal part of the long arm of chromosome 1. Am J Med Genet.

[6] Nuño-Arana I, González-García JR, García-Cruz D (2001). Further clinical delineation in trisomy 1q32 syndrome. Annales de genetique.

[7] Liu M, Ren Y, Wang X, Lu X, Li M, Kim YM (2020). Two rare cases of acute myeloid leukemia with t (8; 16)(p11. 2; p13. 3) and 1q duplication: case presentation and literature review. Mol Cytogenet.

[8] Loh TJ, Lian DW, Iyer P, Lam JC, Kuick CH, Aung AC (2014). Congenital GATA1-mutated myeloproliferative disorder in trisomy 21 complicated by placental fetal thrombotic vasculopathy. Hum Pathol.

[9] Kim WK, Lee NM, Lim IS, Chae SA, Yun SW, Yi DY (2022). Developmental delay and rehabilitation in an infant with partial trisomy 1q32. 1 to 1q44: a case report. Neonatal Med.

